# Overwork and Sanitation in Public Schools

**Published:** 1887-01

**Authors:** 


### *OVERWORK AND SANITATION IN PUBLIC SCHOOLS.*

In recent numbers of the *Annals of Hygiene* appears a series of articles by Dr. Charles K. Mills, on "Overwork and Sanitation in the Public Schools of Philadelphia, with Remarks on the Influence of Overwork in the Production of Nervous Diseases and Insanity." These articles embody the substance of a lecture delivered before the Teacher's Institute of Philadelphia, and of a paper read at the State Sanitary Convention, held in Philadelphia, in May, 1886. Dr. Mills first gives the results of some personal investigations in the Philadelphia public schools. He found the system in a state of more or less confusion, the schools of different grades working under systems or methods which do not dovetail one into the other. Investigations were made in several grammar schools, and in the Central High School and the Normal School. The inquiries made included the number and kinds of studies, the number of hours of school work, of home work, and of sleep; and also as to whether the pupils suffered from any form of ill-health that could be fairly said to be due to school studies. The results show that some overwork occurs, but that improvements have taken place in recent years, the children in not a few places suffering from bad methods of work rather than from genuine overwork. The overwork is particularly marked in some of the girl's grammar schools and in the Normal School. In some schools fewer subjects should be taught. The headache, nervousness, eye-strain, dyspepsia, forms of insanity, and other affections attributed to overwork are discussed in these papers.

Dr. Mills does not advocate a school system without examinations, but believes that the systems should be arranged so that the time will be sufficient to allow each grade to be brought carefully and naturally up to certain standards; in which case an examination, with a minimum average in every branch, would be desirable.

Some investigations were made into the ventilation of Philadelphia schools. It was generally found to be bad, and sometimes execrable. The temperatures of the rooms were almost uniformly a few degrees higher than they should have been. Opportunities for exercise are, as a rule, not properly afforded, and the drainage is, in some instances, very bad.

A system of medical superintendence of the public schools is advocated, so that questions of ventilation, lighting, seating, drainage, vaccination, and attendance of children at whose homes contagious diseases prevails, would come directly under the jurisdiction of a medical director or superintendent. By such inspector or superintendent special investigations could also be made or directed, from time to time, with reference to the number of studies, hours of home work, and amount of recreation and sleep. Attention is strongly called to the fact that, if school children are sometimes overworked, it may be, to a large extent, the fault of their parents. Parents, for instance, should see to it that their girls are not driven too hard during the menstrual periods.

The influence of overwork, in the production of nervous diseases and insanity, is discussed from the standpoint of the effects of special forms of physical overwork upon the nervous system. It is shown that in certain so-called "fatigue disorders," writers' cramp and artizans' diseases, it is the continuous monotonous repetition of the forms and processes of work which brings about the results; and it is held that it is in a consideration of facts of this kind that the true philosophy of neurasthenia, or the philosophy of a true neurasthenia, becomes evident.

